# Complete genome analysis of *Serratia marcescens* RSC-14: A plant growth-promoting bacterium that alleviates cadmium stress in host plants

**DOI:** 10.1371/journal.pone.0171534

**Published:** 2017-02-10

**Authors:** Abdur Rahim Khan, Gun-Seok Park, Sajjad Asaf, Sung-Jun Hong, Byung Kwon Jung, Jae-Ho Shin

**Affiliations:** 1 School of Applied Biosciences, College of Agriculture and Life Sciences, Kyungpook National University, Daegu, Republic of Korea; 2 Department of Plant and Microbial Biology, University of California, Berkeley, CA, United States of America; Universite Paris-Sud, FRANCE

## Abstract

*Serratia marcescens* RSC-14 is a Gram-negative bacterium that was previously isolated from the surface-sterilized roots of the Cd-hyperaccumulator *Solanum nigrum*. The strain stimulates plant growth and alleviates Cd stress in host plants. To investigate the genetic basis for these traits, the complete genome of RSC-14 was obtained by single-molecule real-time sequencing. The genome of *S*. *marcescens* RSC-14 comprised a 5.12-Mbp-long circular chromosome containing 4,593 predicted protein-coding genes, 22 rRNA genes, 88 tRNA genes, and 41 pseudogenes. It contained genes with potential functions in plant growth promotion, including genes involved in indole-3-acetic acid (IAA) biosynthesis, acetoin synthesis, and phosphate solubilization. Moreover, annotation using NCBI and Rapid Annotation using Subsystem Technology identified several genes that encode antioxidant enzymes as well as genes involved in antioxidant production, supporting the observed resistance towards heavy metals, such as Cd. The presence of IAA pathway-related genes and oxidative stress-responsive enzyme genes may explain the plant growth-promoting potential and Cd tolerance, respectively. This is the first report of a complete genome sequence of Cd-tolerant *S*. *marcescens* and its plant growth promotion pathway. The whole-genome analysis of this strain clarified the genetic basis underlying its phenotypic and biochemical characteristics, underpinning the beneficial interactions between RSC-14 and plants.

## Introduction

Plants harbor a diverse community of endophytic bacteria that live in close association, without triggering the disruption or apparent impairment of hosts [1]. In such symbiotic associations, both partners, i.e., the microorganism and host plant, benefit by the mutual interaction with respect to overall fitness. Endophytic bacteria improve host plant growth via phytohormone and siderophore production, nitrogen fixation, and phosphate solubilization or by induced systematic resistance, competition for nutrients, and protection against abiotic stress and pathogens [2]. These mechanisms are of prime importance for plants that are used for large-scale biomass production, especially in phytoremediation. Bacterial endophytes exhibit broad diversity; representative genera include *Bacillus*, *Pseudomonas*, *Azospirillum*, *Burkholderia*, and *Serratia*, and colonize different plant organs [3, 4, 5].

Plant-associated *Serratia* comprise both endophytes and free-living species in the rhizosphere. Many *Serratia* species have plant growth-promoting (PGP) ability and are biocontrol agents for soil-borne fungal pathogens that infect various crops [6, 7]. The production of the phytohormone indole-3-acetic acid (IAA) may be involved in plant growth promotion. *Serratia plymuthica* AS12 and *S*. *plymuthica* AS13 isolated from the roots of rapeseed plants promote host plant growth [8, 9]. Similarly, *S*. *plymuthica* HRO-C48 has been used as a successful biocontrol agent against soil-borne fungal diseases in strawberries and rapeseed [7]. *S*. *marcescens* strain 90–166 was isolated and selected based on its potential as a biological control agent against *Rhizoctonia solani* on cotton [10]. The strain elicits induced-systemic resistance (ISR) against diverse plant pathogens, such as *Colletotrichum orbiculare* [11], cucumber mosaic virus [12], *Erwinia tracheiphila* [13], *Pseudomonas syringae* pv. *lachrymans* [14], and *Fusarium oxysporum* [15].

Under both natural and agricultural conditions, plants are exposed to diverse abiotic and biotic factors [16]. Potentially hazardous heavy metals released into the environment through natural and anthropogenic activities represent a challenge for environmental restoration [17]. Integrated plant microbes have been utilized for enhanced phytoextraction of heavy metal contaminants from the soil in the last decade, with fruitful results [18, 19]. An endophytic bacterial strain, *Serratia nematodiphila* LRE07, has been isolated from *Solanum nigrum* [20]. The strain is Cd-resistant, solubilizes phosphate, and produces IAA and siderophores. Furthermore, the strain promotes host plant growth under Cd contamination. The effective application of such potent bacterial strains to the remediation of contaminated sites requires knowledge of the genetic pathways or resistance mechanism and biotransformation potential within microbial communities. However, the bacterial mechanisms underlying heavy metal detoxification and resistance have not been examined in detail.

Recent advances in next-generation sequencing technologies have increased our opportunities to sequence the complete genomes of beneficial organisms and to characterize their total gene content, genome structure, physiology, ecology, and evolution [21, 22, 23]. In addition, genome annotation, *in silico* biochemical pathway construction, and comparative genome analyses have enabled us to obtain an in-depth understanding of microbial physiology and morphology [24]. Several studies have focused on comparative analyses of endophytes that possess PGP ability [25]. Recently, the genome of the rhizobacterium *S*. *marcescens* 90–166 has been sequenced and many PGP- and ISR-related genes have been identified [26]. Next-generation sequencing methods have been employed to study the genomes of several PGP rhizobacteria belonging to the genus *Serratia*, and these have mainly been isolated as plant endophytes. Many *Serratia* isolates exhibit heavy metal tolerance and have phytoremedial effects on host plants [3, 20, 27]. However, thus far, the complete genome sequences of rhizobacteria with metal-tolerant and PGP properties have not been reported.

Plant growth promotion and enhanced metal phytoextraction in host plants by endophytic bacteria are well-known phenomena; however, the underlying mechanism involved in plant-microbe-heavy metal interactions is poorly understood. Genetic diversity and divergence, ecological adaptations, and horizontal gene transfer among species are factors in these interactions. *S*. *marcescens* RSC-14 was isolated from the Cd hyperaccumulator *Solanum nigrum*. A previous analysis revealed that the strain harbors Cd-tolerance genes and exhibits phosphate solubilization and phytohormone production traits [3]. In the current study, we report the complete genome sequence of the Cd-tolerant PGP strain *S*. *marcescens* RSC-14 to elucidate the genetic traits involved in metal tolerance and plant growth promotion and to define the primary characteristics responsible for endophytism.

## Materials and methods

### Bacterial culture conditions

*S*. *marcescens* RSC-14 was previously isolated from the surface-sterilized roots of *S*. *nigrum*. The strain promotes host plant growth under Cd contamination [3]. Additionally, the microbe has a high tolerance for Cd and is able to solubilize inorganic phosphate and produce phytohormones. The bacterial strain RSC-14 was streaked on a Tryptic Soy Agar plate and incubated at 28°C for 24 h. A single colony was inoculated in 3 mL of Tryptic Soy Broth in a shaking incubator at 28°C for 6 h.

### Genomic DNA isolation, sequencing, and genome assembly

Genomic DNA was extracted from RSC-14 culture broth after 6 h using the Qiagen DNA Mini Kit (Hilden, Germany), according to the manufacturer’s instructions. The DNA concentration was determined using a NanoDrop 8000 UV-Vis Spectrophotometer (Thermo Scientific, Dreieich, Germany). The quality and integrity of extracted DNA were checked by electrophoresis on a 0.8% agarose gel. The DNA was stored at -70°C. A DNA library was constructed according to the manufacturer’s protocol. The complete genome was sequenced using a single-molecule real-time (SMRT) sequencing platform on a PacBio RS II instrument with P6-C4 chemistry (Pacific Biosciences, Menlo Park, CA, USA) [28]. In total, 66,588 raw reads were generated with an average length of 11,968 bp using two SMRT Cells. They were *de novo* assembled using the hierarchical genome-assembly process (HGAP) [29] protocol RS HGAP Assembly 2 implemented in SMRT Analysis version 2.3 (Pacific Biosciences; https://github.com/PacificBiosciences/SMRT-Analysis). Standard parameters were applied, including PreAssembler v2, Minimum Seed Read Length 6,000, Celera Assembler v1, Genome Size (Bp) 5,000,000, Target Coverage 30, Overlapper Error Rate 0.06, Overlapper Min Length 40, and Overlapper K-mer 14. The complete genome was generated with 155-fold coverage (59.61% G+C).

### Genome annotation

The *S*. *marcescens* RSC-14 genome was functionally annotated using the NCBI Prokaryotic Genomes Automatic Annotation Pipeline (PGAAP), which uses Glimmer 3.0 for the identification of protein-coding genes, tRNAscan-SE for tRNA genes, and RNAmmer for rRNA genes. Functional annotation was carried out using the Rapid Annotation using Subsystem Technology (RAST) server with the seed database [30]. The pipeline (PGAAP) uses GeneMark to predict open reading frames (ORF) and searches against Protein Clusters. Protein-coding genes were searched against the NCBI RefSeq database using BLASTp. The ORFs were assigned to COG functional categories by BLAST searches against the COG database. InterPro searches were also performed to identify conserved domains in each ORF. An average nucleotide identity (ANI) analysis was performed using all complete genome sequences of the *Serratia* genus available in the EzBioCloud database (http://www.ezbiocloud.net/eztaxon). Metabolic pathways were constructed based on the KEGG (Kyoto Encyclopedia of Genes and Genomes) database. A local BLASTP search was carried out for genes of interest. Reciprocal BLAST was performed for a Pan Genome analysis of the five *Serratia* spp. using the web-based program to determine shared orthologs. For prophage identification, PHAST (PHAge Search Tool; http://phast.wishartlab.com/) was used. For potential genome rearrangement detection, the genomes of *Serratia* spp. were aligned using Mauve with default parameters and the Progressive Mauve option. Pan genome analysis was performed using OrthoVenn, a web platform to compare and annotate orthologous gene clusters among multiple *Serratia* species (http://aegilops.wheat.ucdavis.edu/OrthoVenn/).

### Phylogenetic analysis

For the comparative phylogenetic analysis, the sequences of the housekeeping loci 16S rRNA, *gyr*B, and *rpo*B of different PGP *Serratia* species and *Photorhabdus luminescens* subsp. *laumondii* TTO1 (as outgroup) were retrieved from NCBI. A phylogenetic tree was constructed based on the concatenated sequences of the three housekeeping genes using the neighbor-joining algorithm in MEGA6. The consensus tree was inferred using 1,000 bootstrap replicates.

### Screening for plant growth-promoting traits

The phosphate solubilization potential of the strain RSC-14 was previously confirmed using NBRIP medium. IAA production in culture media has also been confirmed by a GC-MS analysis [3]. The strain was tested for siderophore production using the chrome azurol S agar plate method, as described by Louden et al. [31]. The plates were incubated for 7–10 days at 30°C. For method confirmation, a laboratory strain *Chryseobacterium lactis* which can produce siderophores was inoculated on a chrome azurol S agar plate. Chitinase, cellulose, and pectin degradation activity were confirmed using a plate assay according to the methods of Tahtamouni et al. [32]. The ability to produce ammonia was evaluated using Nessler’s reagent method. The nitrogen fixation ability of the strain was qualitatively evaluated based on the appearance of bacterial growth on nitrogen-free medium. The experiment was performed in triplicate.

### Plant inoculation experiment

Surface-sterilized seeds were germinated in sterile petri dishes lined with moistened Whatman filter paper number 2, as described by Khan et al. (2016). After seed germination for 1 week, equally sized seedlings (5 per Petri dish) were transferred to new sterilized Petri dishes containing a sterile Whatman filter paper moistened with sterile distilled water. The RSC-14-inoculated treatment received 5 mL of an RSC-14 suspension with an optical density at 600 nm (OD_600_) of 0.5. The seedlings were allowed to grow in a growth chamber with a 14-h photoperiod (350 μmol m^−2^ s^−1^) at 25°C and 60% relative humidity for 15 days, and the root/shoot lengths were measured on the final day of harvesting.

## Results

### Genome sequencing statistics and genome features

**Fig 1:**
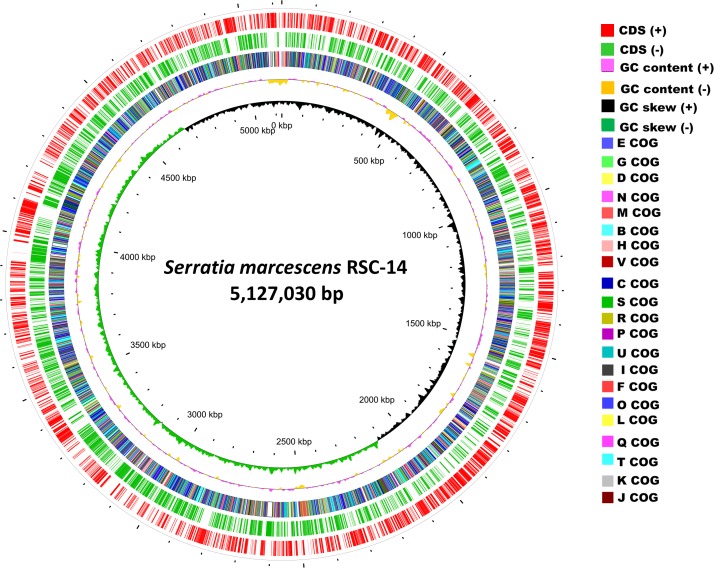
Graphical circular genomic maps of *Serratia marcescens* RSC-14. The two outer circles show the predicted protein-coding sequences on the plus (green) and minus (blue sky) strands. The third circle shows the distribution of genes related to COG categories. The fourth circle shows the GC contents, and the fifth circle shows the GC skew

The genome was sequenced using the SMRT system with an output of 66,588 reads. The mean read length was 11,968 bp and the longest read was 49,588 bp. The complete genome of *S*. *marcescens* RSC-14 comprises a single circular chromosome of approximately 5.12 Mbp (175% coverage), with an overall G+C content of 59.61% (Fig 1). General characteristics of the complete genome are summarized in Table 1. It contains 4,593 predicted protein-coding genes, 22 rRNA genes, 88 tRNA genes, and 41 pseudogenes. The RSC-14 strain contains no plasmid. The whole genome shotgun project has been deposited at DDBJ/EMBL/GenBank under the accession NZ_CP012639.1. Genomes comparison between *S*. *marcescens* RSC-14 and other strains of the genus *Serratia* revealed that the strain RSC-14 has a size of genome comparable to other *Serratia* species, but a slightly smaller genome (5.12 Mbp) than that of WW4 (5.24 Mbp). Some prophage regions and red pigment biosynthesis gene cluster are absent from the RSC-14 genome. Higher GC% contents were observed for the *S*. *marcescens* genomes than the genomes of other *Serratia*.

**Table 1:** Comparative genome statistics of *S*. *marcescens* RSC-14 and other *Serratia* spp.

	*S*. *marcescens*			*S*. *plymuthica*	*S*. *liquefaciens*
	RSC 14	WW4	FGI94	S13	ATCC27592
Genome size (bp)	5,127,030	5,241,455	4,858,216	5,467,306	5,238,612
GC contents	59.6	59.6	58.9	56.2	55.4
CDS	4,593	4,764	4,328	4,905	4,769
Genes	4,745	4,868	4,436	5,013	4,867
rRNA	22	22	22	22	19
tRNA	88	81	83	85	78
No of plasmid	0	1	0	0	1
No of chromosome	1	1	1	1	1
Site of isolation	Plant [this study]	Paper machine [35]	Fungus garden [34]	Plant [36]	Milk [68]

The genome features for each *Serratia* strain were retrieved from the NCBI data base (http://www.ncbi.nlm.nih.gov/sites/genome/).

### Taxonomic classification

**Fig 2:**
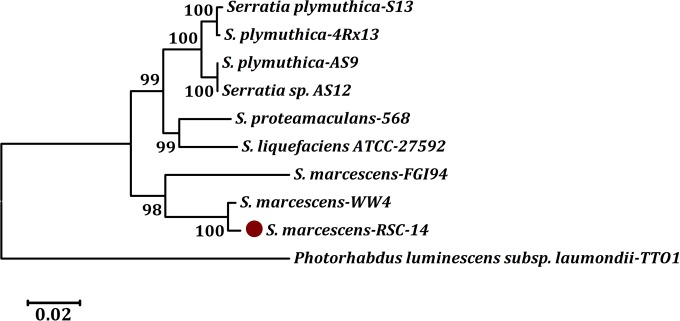
Phylogenetic tree highlighting the position of *S*. *marcescens* RSC-14 with other closely related species within the genus of *Serratia*. The phylogenetic tree was constructed based on concatenated sequences of 16S rRNA, *gyr*B, and *rpo*B genes aligned in ClustalW2 using the neighbor-joining algorithm in CLC Main Workbench and rooted with *Photorhabdus luminescens subsp*. *laumondii* TTO1. All *Serratia* species clustered together and were distinct from other Enterobacteriaceae. The tree also highlights the close relationship of *S*. *marcescens* RSC-14 strain with the *S*. *marcescens* type strain WW4.

The bacterial strain RSC-14 was previously isolated from *S*. *nigrum* and identified based on partial 16S rRNA gene sequences [3]. The complete 16S rRNA gene sequence of the RSC-14 genome was searched against the most recent GenBank NCBI database using BLAST with default settings. The relative frequencies of closely related taxa and BLAST scores were obtained. *Serratia* was the most frequent genus obtained in the BLAST search, and some ‘hits’ for species in this genus shared 100% sequence identity. When considering high-scoring segment pairs from the best 250 hits among *Serratia* spp., the most frequent matches were unspecified *Serratia* strains (25%), which had a maximum identity of 99–100%, and 75% were *S*. *marcescens*, which had a maximum identity of 99–100%. To better define the taxonomic classification of strain RSC-14, three core housekeeping genes, 16S rRNA, *gyr*B, and *rpo*B, which are conserved among bacteria, were examined in a phylogenetic analysis. A phylogenetic tree based on the concatenated sequences of the three genes showed that *Serratia* RSC-14 is closely related to all *Serratia* spp. Two sister clades of *Serratia* clustered together and were distinct from *P*. *luminescens* subsp. *laumondii* TTO1. RSC-14 clustered with *S*. *marcescens* WW4, and this group was supported by a high bootstrap value of 100% (Fig 2). Based on the ANI analysis with all complete genome sequences of *Serratia* spp., *S*. *marcescens* RSC-14 was most similar to *S*. *marcescens* strain 90–166 (ANI value, 95.6%).

### Morphology and physiology

**Fig 3:**
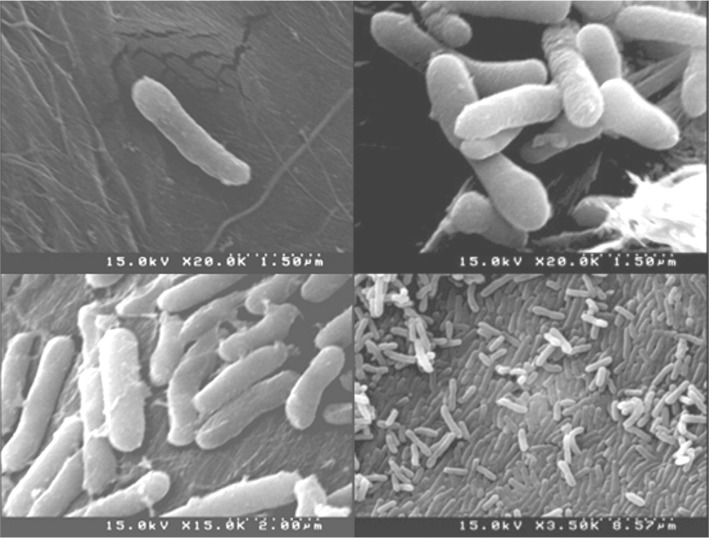
Scanning electron micrograph of *S*. *marcescens* RSC-14. Fatty acid analysis indicated that the sum of C:14 3OH (3.8%), C16:0 (29.8%), C16:1 (8.2%) and C18:1 (5%) in RSC-14 is about 50%, which is consistent with the ratio of 50 to 80% observed in other *S*. *marcescens* strains [33]

*S*. *marcescens* RSC-14 is a Gram-negative, rod-shaped bacterium (Fig 3) with an optimal growth temperature of 28°C.

### Comparative genome analysis

**Fig 4:**
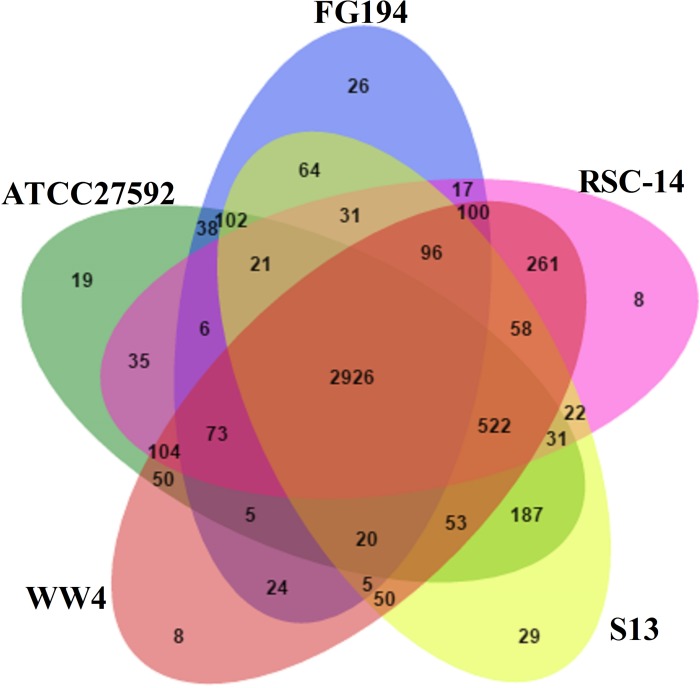
Venn diagram of the CDSs shared by the genomes of five closely related *Serratia* spp.; *S*. *marcescens* FGI94 (CP003942), *S*. *marcescens* WW4 (CP003959), *S*. *liquefaciens* ATCC27592 (CP006252) and *S*. *plymuthica* S13 (CP006566). The number outside the overlapping regions represents the number of CDSs present in each genome without homologs in other *Serratia* strains. While the overlapping regions indicates the CDSs shared by the respective strains.

The *Serratia* strains shared most genes assigned to general cellular functions. As shown in the Venn diagram constructed for five representative *Serratia* genomes in Fig 4, all strains shared 2926 CDSs. The strain RSC-14 shared the most (261) additional CDSs with *S*. *marcescens* WW4, aside from the core collection of genes (Fig 4). The fewest unique CDSs (8) were detected in both *S*. *marcescens* RSC-14 and *S*. *marcescens* WW4, while *S*. *plymuthica* S13 had the highest number of unique genes.

Most of the unique genes in the genomes of each strain were classified as hypothetical proteins and were associated with prophages. The RSC-14 strain had two prophage regions, including one intact prophage and one partial prophage region (S1 Fig). In WW4, there were six prophage regions, contributing a larger proportion of the total genome length. A total of 12 putative prophage regions across the five *Serratia* genomes were detected. Orthologs are genes in different species that evolved from a common ancestral gene via speciation and most retain the ancestral function during evolution. The five *Serratia* species had 4991 clusters and 4901 ortholog clusters that included at least two species. To better understand the relatedness among taxa based on the genomes, a MAUVE progressive alignment was performed for RSC-14 with other genomes selected based on the phylogenetic tree of concatenated genes. *S*. *marcescens* RSC-14 had a similar gene cluster distribution to that of WW4, supporting the assignment of strain RSC-14 to *S*. *marcescens* (data not shown). RSC-14 was previously isolated from surface-sterilized roots of *S*. *nigrum* (Table 1), but was more closely related in the phylogenomic analysis to *S*. *marcescens* WW4, a biofilm-forming bacterium isolated from paper machine aggregates [34] than to plant-associated *S*. *plymuthica* S13, which was isolated from the anthosphere of *Cucurbita pepo* L., but was also able to colonize the seeds, endosphere, and rhizosphere of the same plant species [35]. A Mauve analysis confirmed that the synteny between *S*. *marcescens* RSC-14 and *S*. *marcescens* WW4 was highly conserved (data not shown). No genomic reorganization was observed between the two strains. In contrast, several chromosomal rearrangements were observed between the S. marcescens RSC-14 and FGI94 genomes. *S*. *marcescens* FGI94 was isolated from the fungus garden of the leaf-cutter ant *Atta colombica* [36].

### Plant growth-promoting traits

The strain was initially characterized by chemical and biological assays for various PGP traits. It produced IAA and solubilized inorganic phosphate. However, RSC-14 lacked the ability to fix nitrogen and to produce ACC deaminase and siderophores. Certain *Serratia* strains, such as *Serratia* sp. LJ-1, have potential applications for the biodegradation of phenolic compounds and ammonium via heterotrophic nitrification-aerobic denitrification [37]. The genome of RSC-14 also included genes (AN479_RS11385 and AN479_RS11390) responsible for denitrification, and this phenotype was confirmed in a plate-based bioassay. PGP rhizobacteria are also important for host plants owing to their antagonistic activities, e.g., chitinase activity, which degrades the fungal wall and insect exoskeletons. Most *Serratia* strains exhibit chitinase activity [38]. However, the chitinolytic trait was not found in RSC-14. The strain had no chitinase, cellulose, nor pectin degradation activity. Interestingly, the genome analysis indicated that the strain has four genes annotated as chitinase. The NCBI blast results indicate that these putative genes have a nucleotide identity of around 96% compared to orthologs of other *Serratia* strains. Therefore, it is presumed that there may be a problem with gene expression, absence of secretion or mutations that could interfere with the correct enzymatic activity.

### Indole-3-acetic acid biosynthesis

**Fig 5:**
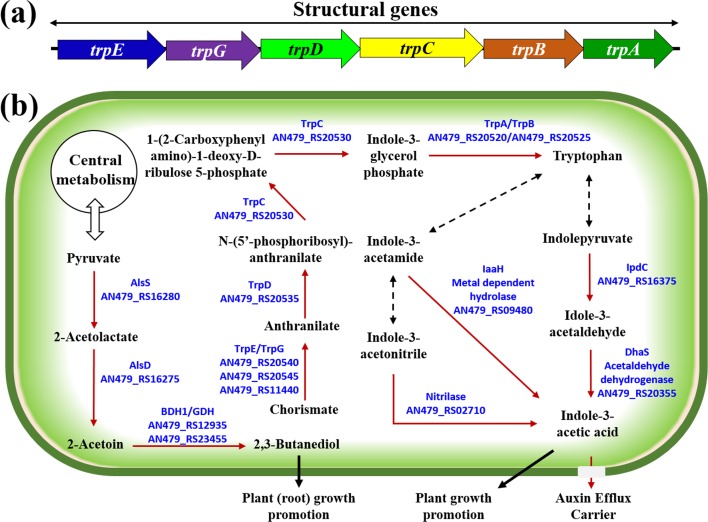
Bacterial metabolic pathways involved in indole-3-acetic acid, tryptophan, and acetoin biosynthesis in *S*. *marcescens* RSC-14. **(a)** Structural gene cluster of the tryptophan biosynthesis pathway. **(b)** The biosynthetic pathways were constructed based on the KEGG database, NCBI genome annotation, and the RAST server. Black shaded arrows indicate known pathway steps for which related genes were not detected in the RSC-14 genome. Red arrows indicate putative enzymes that are encoded by the genome. The enzymes that were identified in the RSC-14 genome are shown with their NCBI locus tag.

In the genomic sequence of RSC-14, we identified genes involved in nutrient availability, oxidative stress resistance, and phytohormone production (Fig 5). The strain promoted host growth based on a seedling bioassay in Petri dishes (S2 and S3 Figs). In a comparative genome analysis, we detected the *trp*EGDCBA gene cluster encoding key enzymes in the tryptophan biosynthesis pathway (Fig 5). Tryptophan synthesis is related to multiple biological processes, including IAA biosynthesis. Endophytic bacteria also promote host plant growth via the synthesis of phytohormones, such as IAA, which is highly produced in various plant-associated endophytes, such as *P*. *putida* W619. IAA synthesis by *S*. *marcescens* strain RSC-14 was confirmed in a previous study using the Salkowski assay and a gas chromatography mass spectrometric analysis [3]. In bacteria, IAA synthesis occurs via three main pathways, two of which are encoded in the genome of strain RSC-14 (Fig 5). The genome sequence of RSC-14 also contained *ipd*C (AN479_RS16375), which encodes indolepyruvate decarboxylase, and *dha*S (AN479_RS20355) which encodes indole-3-acetaldehyde dehydrogenase. Both gene products are needed for the production of IAA from tryptophan. The pathway involves tryptophan-2-monooxygenase (IaaM), which oxidizes tryptophan to indole-3-acetamide, and an indoleacetamide hydrolase (IaaH) that produces IAA. In strain RSC-14, a putative *iaa*H gene (AN479_RS09480) was present, but *iaa*M was not found. The RSC-14 genome also had a gene involved in one pathway to synthesize IAA via indole-3-acetaldehyde which is converted from indolepyruvate by indolepyruvate decarboxylase (IpdC, AN479_RS16375) In addition to these genes in the pathway for IAA production, a putative gene encoding an auxin-binding protein (AN479_RS22585) was also identified in the genome of RSC-14.

### Acetoin and butanediol synthesis

In addition to IAA, volatile compounds released by rhizobacteria, such as 3-hydroxy-2-butanone (acetoin) and 2,3-butanediol, can enhance plant growth [39, 40]. Acetolactate synthase (AlsS) and acetolactate decarboxylase (AlsD) catalyze the two-step conversion from pyruvate to acetoin. Subsequently, acetoin can be converted to 2,3-butanediol, either by the bacteria or by the host plant. The *alsDS* acetoin synthesis pathway was previously detected in *S*. *proteamaculans* 568 [41]. The *S*. *marcescens* strain RSC-14 genome carries genes encoding acetolactate synthase (AN479_RS10660) and acetolactate decarboxylase (AN479_RS16275), which convert small fractions of pyruvate to acetoin.

### Phosphate solubilization

The mineral phosphate solubilization potential of *S*. *marcescens* RSC-14 was previously confirmed using chemically defined medium containing insoluble phosphate [3]. The major mechanism for phosphate solubilization in bacteria is the synthesis and secretion of gluconic acid [42]. The production of gluconic acid requires glucose dehydrogenase and its cofactor pyrroloquinoline (PQQ) [43]. The gene encoding PQQ-dependent glucose dehydrogenase and the operon consisting of *pqq*ABCDEF, required for PQQ biosynthesis, were identified in the genome of RSC-14 (S4 Fig). Additionally, other relevant genes, such as those encoding lactate dehydrogenase (EC 1.1.1.28), citrate synthase (EC 2.3.3.1), inorganic pyrophosphatase (EC3.6.1.1), and alkaline phosphatase (EC 3.1.3.1) were found in the genome of RSC-14 (Table 2).

**Table 2:** Genes (and their locus tags) involved in phosphate solubilization.

Locus_tag	Activity		Gene product
AN479_RS07990	Phosphate metabolism	Inorganic pyrophosphatase (EC 3.6.1.1)	
AN479_RS12945	Phosphate metabolism	Alkaline phosphatase (EC 3.1.3.1)	
AN479_RS02355	Oxidation of malate to oxaloacetate	Malate dehydrogenase (EC 1.1.1.37)	
AN479_RS06600	Oxidation of 3-Isopropylmalate to 4-methyl-2-oxopentanoate, 2-isopropyl-3-oxosuccinate, or 4-methyl-2-oxopentanoate	3-Isopropylmalate dehydrogenase (EC 1.1.1.85)	
AN479_RS18465	Oxidation of beta-D-glucose to D-glucono-1,5-lactone	Glucose 1-dehydrogenase (EC 1.1.1.47)	
AN479_RS03395	Oxidation of (R)-lactate to pyruvate	d -Lactate dehydrogenase (EC 1.1.1.28)	
AN479_RS14060	Oxidation of (S)-lactate to pyruvate using 2 ferricytochrome	l -Lactate dehydrogenase (EC 1.1.2.3)	
AN479_RS02845	Synthesis (2S,3S)-2-hydroxybutane-1,2,3-tricarboxylate from Propanol-CoA and Oxaloacetate	2-Methylcitrate synthase (EC 2.3.3.5)	

Note: Genes were identified using KEGG, RAST, and NCBI genome annotations; locus tags represent the gene in the NCBI database, and functions are based on data obtained from the RAST server.

### Prodigiosin biosynthesis

Prodigiosin is a red-pigmented produced by various bacteria, such as *Actinobacteria*, *Serratia*, and some marine bacteria. However, the strain RSC-14 forms white colonies. Compared to *S*. *marcescens* WW4, the strain RSC-14 lacks the complete *pig* cluster for the biosynthesis of prodigiosin. The *pig* cluster of *S*. *marcescens* is flanked by *cue*R and *cop*A, which are involved in copper-dependent regulation and efflux. In the RSC-14 genome, *cue*R (AN479_RS04580) and *cop*A (AN479_RS04585) are separated by 107 bp. Several antibiotic resistance related genes were also annotated and listed in S1 Table.

### Cadmium tolerance and oxidative stress response

In the RSC-14 genome, many genes responsible for Cd tolerance and transportation were detected. Three genes encoding lead/cadmium/zinc/mercury-transporting ATPase as well as three putative genes encoding the cobalt/zinc/cadmium resistance protein CzcD were identified; these loci are predicted to be involved in Cd tolerance and transportation (S1 Table). Additionally, five genes encoding putative arsenic (As)-resistance related proteins were found in the genome. As-related genes encode an arsenical efflux pump membrane protein (AN479_RS18760), an arsenical resistance operon repressor (AN479_RS18755), and three arsenate reductases (EC 1.20.4.1, AN479_RS15850, AN479_RS15965, AN479_RS18765). In a genome analysis using the RAST server and NCBI annotation, several enzymes involved in the oxidative stress response were identified (S1 Table). The genome of RSC-14 encodes three superoxide dismutases: SodA, a Mn superoxide dismutase (AN479_RS09560), SodB, a Cu/Zn superoxide dismutase (AN479_RS22680), and SodC, a Fe superoxide dismutase (AN479_RS22745). It also encodes a catalase (EC 1.11.1.6) (AN479_RS17090) and one peroxidase (EC 1.11.1.7) (AN479_RS17790), which have predicted functions in oxidative stress and protection from reactive oxygen species (ROS).

## Discussion

Phytohormone production is the most common feature shared by beneficial bacterial endophytes [44, 45, 46, 47, 48]. Plant-associated endophytes utilize tryptophan in the plant exudate as a precursor for the biosynthesis of IAA [45]. The host PGP ability of endophytic bacteria is attributed to their IAA production. Host plants are thought to select endophytes that have the IAA production trait from the rhizosphere, and this selection process might explain the higher prevalence of IAA-synthesizing bacteria inside plant tissues than in the rhizosphere [45]. Various isolates of *S*. *marcescens* promote host plant growth and provide protection against fungal pathogens [10, 38, 49]. Many isolates, such as *S*. *marcescens* NBR11213 and *S*. *marcescens* TRS-1, have phosphate solubilization ability, produce IAA, and are antagonistic to fungal pathogens [38, 49, 50]. *S*. *marcescens* CDP-13 has various PGP traits, such as the production of IAA and ACC deaminase, phosphate solubilization, nitrogen fixation, and ammonia production. The isolate alleviates salt stress in wheat, promotes plant growth, and strengthens plant immune responses [51].

IAA production by *S*. *marcescens* strain RSC-14 has been detected previously[3], we analyzed the RSC-14 genome for the presence of genes involved in IAA biosynthesis and found genes involved in IAA production from indole-3-acetamide as well as from indole-3-pyruvate (Fig 5). Two enzymes, indolepyruvate decarboxylase (AN479_RS16375) and acetaldehyde dehydrogenase (AN479_RS20355), are involved in the conversion of tryptophan to IAA via indolepyruvate. In this pathway, tryptophan is first converted to indolepyruvate, which is subsequently converted to indole-3-acetaldehyde via indolepyruvate decarboxylase and then to IAA via acetaldehyde dehydrogenase. Our results are in agreement with previous studies of the genomes of several endophytes, such as *Enterobacter* from poplar stems [52], *Gluconacetobacter diazotrophicus* from sugarcane [53], and the endophyte community of rice [54]. Our results revealed that the genome of *S*. *marcescens* RSC-14 carries many genes that may be useful for integrated bioremediation and that may specifically improve the growth of hyperaccumulator plants, such as *S*. *nigrum*. The strain can stimulate plant growth directly via the production of phytohormones, such as IAA.

Root elongation depends on the production of acetoin or via 2,3-butanediol; root growth is significantly greater in RSC-14-inoculated plants than in non-inoculated control plants [3]. Acetoin and butanediol are two well-known volatile compounds that act as growth-promoting factors and also increase plant resistance against pathogens [55, 56, 57, 58]. Additionally, the increased production of these beneficial compounds results in enhanced root growth and development, thus improves access to nutrients and water and consequently increases the establishment of host plants in extreme conditions, such as abiotic stress conditions. Beside that, acetoin and 2,3-butanediol production by some PGP bacteria induces systemic resistance and drought tolerance [10]. These findings are consistent with our previous observations that *S*. *marcescens* RSC-14 increases growth attributes in *S*. *nigrum* under Cd contamination [3]. A similar growth-promoting effect has also been observed for *S*. *nematodiphila* LRE07, which increases the tiller ratio and fresh biomass in *S*. *nigrum* plants under Cd stress [3, 20, 27]. *S*. *marcescens* strain 90–166 is a potential biological control agent against *Rhizoctonia solani* on cotton [10]. It confers ISR against *Colletotrichum orbiculare* [11], cucumber mosaic virus [12], *Erwinia tracheiphila* [13], *Pseudomonas syringae* pv. *lachrymans* [14], and *Fusarium oxysporum* [15]. Some genes involved in the metabolic pathway of acetoin and butanediol synthesis were detected in the RSC-14 genome. The biosynthesis pathway of acetoin and butanediol starts from the intermediate metabolite pyruvate, which is produced during central metabolic processes. Pyruvate could be converted to 2-acetolactate via a putative acetolactate synthase (AN479_RS16280) and be further decarboxylated into 2-acetoin via acetolactate decarboxylase AlsD (AN479_RS16275).

The interconversion between acetoin and 2,3-butanediol requires a 2,3 butanediol dehydrogenase (BDH) activity. Three BDHs and one glycerol dehydrogenase (GDH) were recently identified in *Serratia* sp. T241 as involved in the formation of different isomers of 2,3-butanediol [58]. The *S*. *marcescens* RSC-14 genome was searched for homologs of the *bdh* and *gdh* genes. We found that the products of locus tags AN479_RS12935 and AN479_RS23455 present 95% and 93% aa sequence identity with the BDH1 and GDH enzymes, respectively. This suggests that the corresponding genes are involved in 2,3-butanediol biosynthesis.Phosphorus (P) is required for plant growth. However, the majority of soil P occurs as a combined mineral with minimal availability for plant uptake. The phosphate-solubilizing ability of microbes has extensive agricultural applications [59, 60, 61]. Phosphate solubilization in microbes is mainly attributed to their production and secretion of low-molecular-weight organic acids, such as gluconic acid, 2-ketogluconic acid, citric acid, oxalic acid, and phosphatases [62]. The phosphate solubilization ability of RSC-14 was confirmed on specific medium supplemented with insoluble phosphate. Genes involved in gluconic acid production or in other low-molecular-weight organic acid production were indeed detected in the RSC-14 genome (S4 Fig).

Cd is a redox-stable metal that mediates oxidative stress via an indirect mechanism by depleting glutathione and protein-bound sulfhydryl groups, which results in the enhanced production of ROS, such as superoxide ions, hydrogen peroxide, and hydroxyl radicals [63, 64]. RSC-14 is resistant to Cd with a minimum inhibitory concentration of 4 mM [3]. The Cd-resistance genes and other heavy metal-resistance genes in the genome of RSC-14 might be involved in Cd uptake, accumulation, and detoxification within the cell. Cd is “look alikes” for Zn; it currently has no known biological function in bacteria and disrupts normal organismal processes. Low-level resistance to Cd/Zn is achieved by binding the metal ions in the inactive form non-specifically to the cell wall. Zn and Cd resistance in bacteria is mainly attributed to the active efflux of these metal ions to prevent toxic effects in the cell. The resistance mechanisms of Cd and Zn are indistinguishable, involving the same genes. The efflux of Cd is facilitated by P-type ATPases, CBA transporters, and CDF chemiosmotic transporters. The P-type ATPases comprise a superfamily of transport proteins associated with the membrane that actively transport ions against the concentration gradient by ATP hydrolysis. Efflux transporters, which play a significant role in heavy metal resistance and homeostasis, are categorized as the P1B-type subfamily of ATPases [65]. There are two additional subgroups, Cu/Ag-translocating and Zn/Cd/Pb-translocating P1B-type ATPases, which are defined based on substrate specificity [66]. The physiological function of these efflux pumps is to maintain the homeostasis of essential metals, including Cu, Zn, and Co. However, they also mediate resistance to other non-essential toxic metals, like Cd, Pb, and Ag [66]. A number of genes encoding Zn ABC transporters, Zn binding proteins, Zn metalloproteases (RseP and HtpX), and P-type ATPase were identified in the RSC-14 genome. The Pb/Cd/Zn/Hg transporting ATPase genes, as well as three genes encoding homologs of the Co/Zn/Cd resistance protein CzcD were identified in the RSC-14 genome; these loci are involved in Cd homeostasis and transportation. Additionally, 5 potential As-resistance genes were also detected in the genome.

Heavy metals, such as Cd, cause oxidative stress, which triggers an increase in the production of antioxidants and enhanced activity of antioxidant enzymes to neutralize Cd-induced toxicity. In eukaryotes, the induction of the antioxidative immune response involves the sequential and simultaneous action of different enzymes, such as superoxide dismutase, catalase, and guaiacol peroxidase, and non-enzymatic scavengers, such as glutathione, ascorbate, carotenoids, and polyphenols, which are responsible for the scavenging of excessive ROS under stress conditions [62, 67]. Several antioxidant enzymes, such as superoxide dismutase, catalase, and glutathione peroxidase, and reduced glutathione produced by RSC-14 may enable plants to tolerate and detoxify Cd. The antioxidative enzymes may also collectively contribute to the alleviation of oxidative stress caused by intracellular Cd accumulation [3].

## Conclusion

In this study, we determined the complete genome sequence of *S*. *marcescens* strain RSC-14 and identified genes related to IAA production, Cd tolerance, and phosphate solubilization. This is the first report of a Cd-tolerant *S*. *marcescens* genome sequence and its potentials pathways involved in PGP activity. The IAA pathway-related genes and oxidative stress-responsive enzyme genes may explain the PGP potential and Cd tolerance of the strain, respectively. Finally, this study provides a foundation for future genetic investigations of this bacterium and its molecular interactions with host plants.

## Supporting information

S1 Fig Predicted prophage regions in *S. marcescens* RSC-14 genome. Two prophage regions were detected in the genome of RSC-14.
(TIF)

S2 Fig Representative seedlings of *Solanum nigrum* at the final day of harvest (15 days). Seedlings labeled 1–3 were treated with water, while those labeled 4–6 were treated with an RSC-14 suspension. The seedlings are representative of 3 independent experiments.
(TIF)

S3 Fig Effect of RSC-14 inoculation on shoot and root lengths of *Solanum nigrum* seedlings on the final day of harvest (15 days). Each value represents the mean ± SE of three replicates per treatment from 3 independent experiments. Asterisks (*) represent significant differences between treatments at *p* < 0.05 calculated by Duncan’s multiple range tests.
(TIF)

S4 Fig Proposed scheme for the gluconic acid synthesis in RSC-14. **Gluconic acid is secreted by bacteria and plays a major role in phosphate solubilization [34].** The first enzyme of the gluconic acid pathway and cofactor (PQQ) are shown in blue. The gene encoding Glucose dehydrogenase and the *pqq*ABCDEF operon were identified in the RSC-14 genome as locus-tags AN479_RS18465 and AN479_21790, AN479_RS21865, AN479_RS21870, AN479_RS21875, AN479_RS20540, AN479_RS21885, respectively.
(TIF)

S1 Table Annotated important genes from the *Serratia marcescens* RSC-14 complete genome. (XLSX)

S2 Table Fatty acid components of the *Serratia marcescens* RSC-14. (XLSX)
