# Supplementary figures and images for: Complete genome analysis of Serratia marcescens RSC-14: A plant growth-promoting bacterium that alleviates cadmium stress in host plants
Source: PLoS One. 2017 Feb 10;12(2):e0171534. doi: 10.1371/journal.pone.0171534 (PMC5302809; doi:10.1371/journal.pone.0171534)

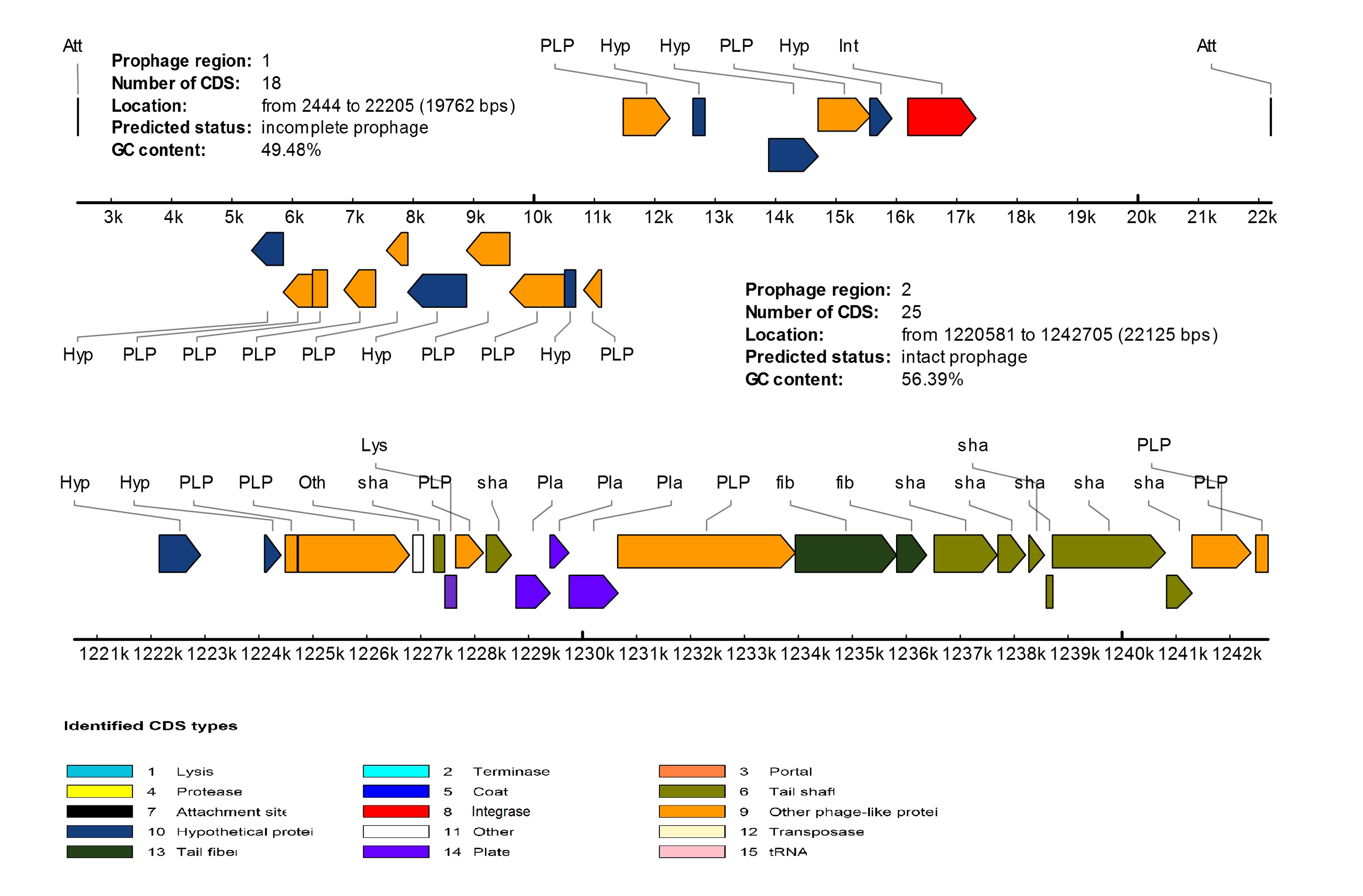

Supplement: S1 Fig — Two prophage regions were detected in the genome of RSC-14. (TIF) [file pone.0171534.s001.tif]

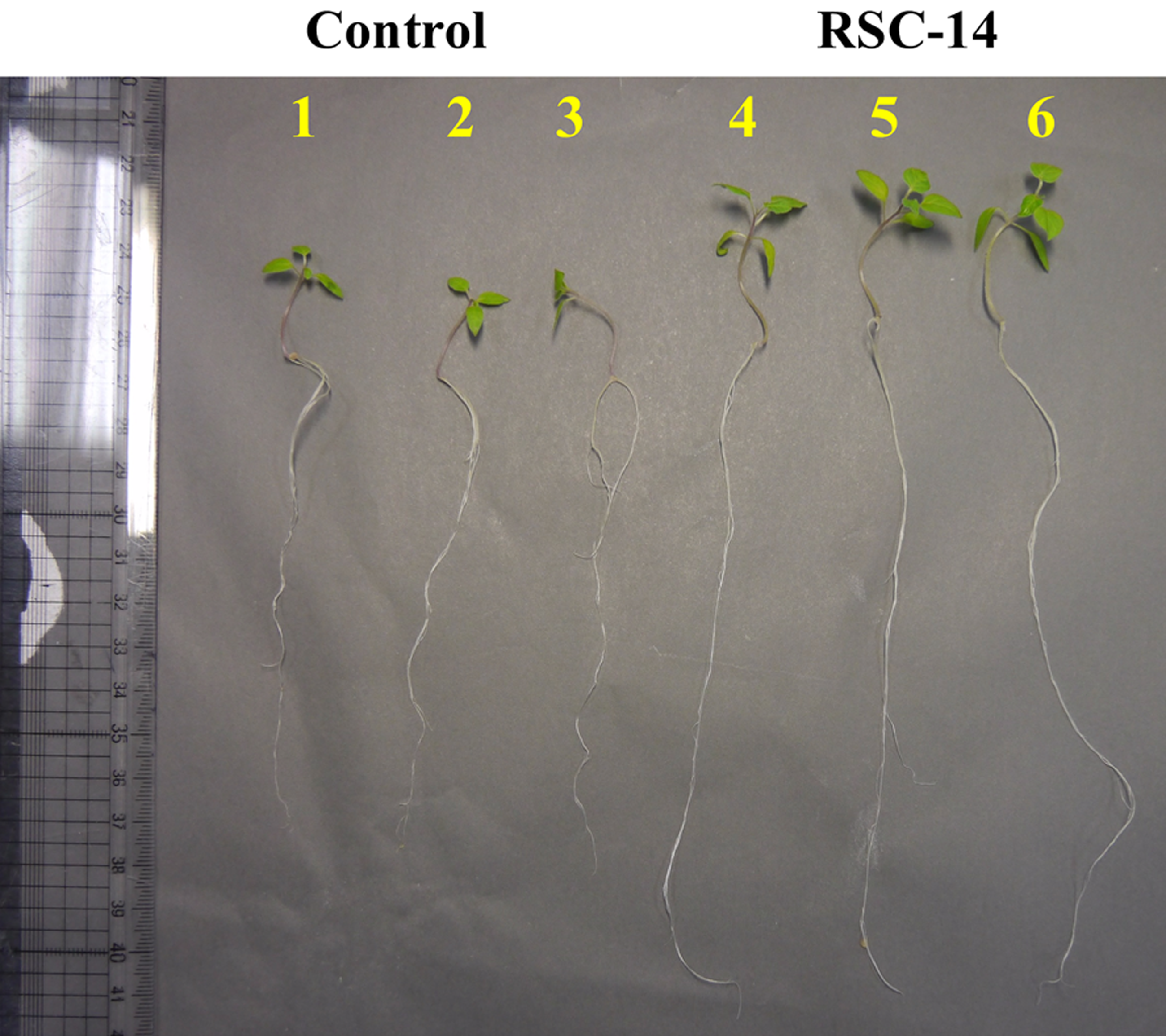

Supplement: S2 Fig — Seedlings labeled 1–3 were treated with water, while those labeled 4–6 were treated with an RSC-14 suspension. The seedlings are representative of 3 independent experiments. (TIF) [file pone.0171534.s002.tif]

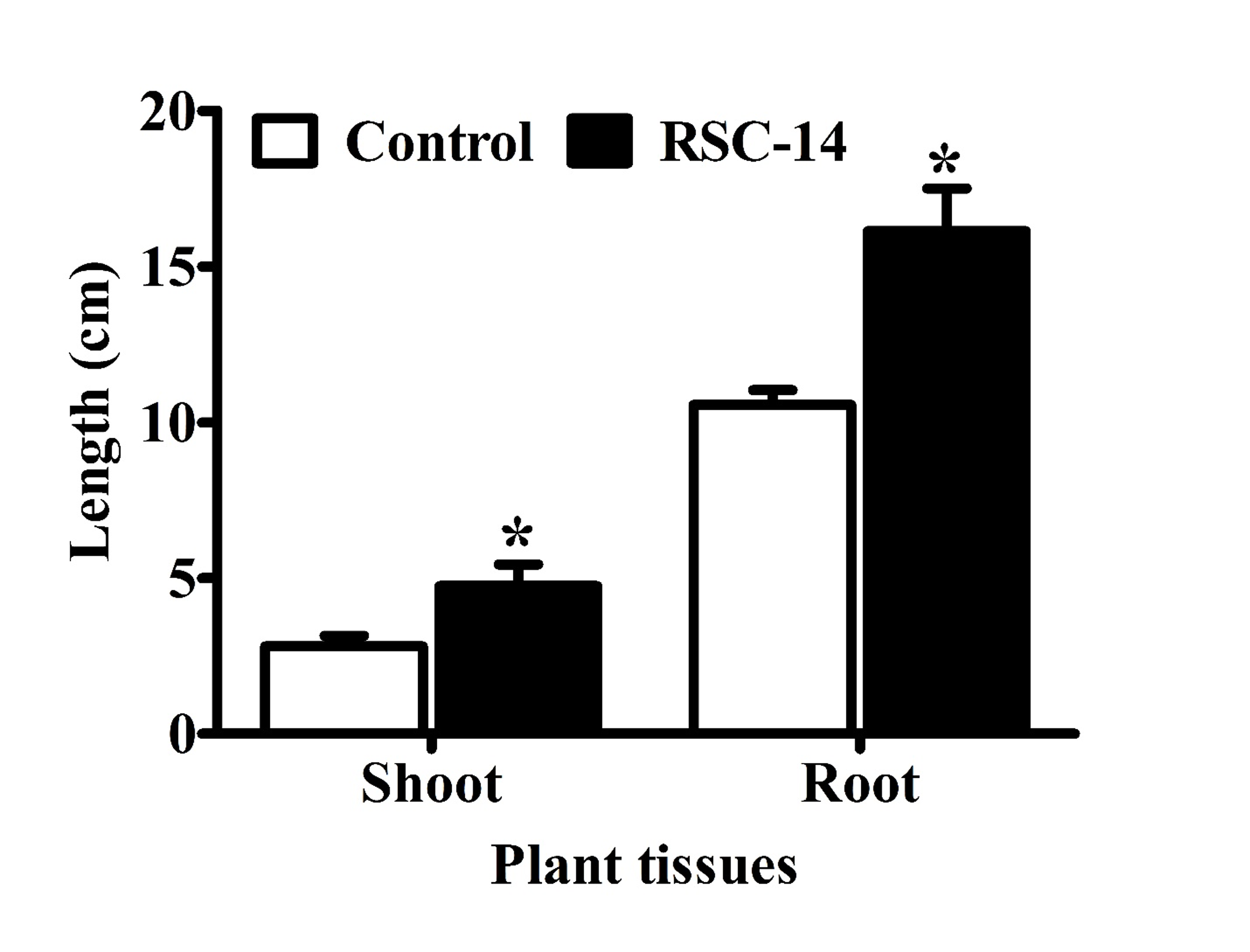

Supplement: S3 Fig — Each value represents the mean ± SE of three replicates per treatment from 3 independent experiments. Asterisks (*) represent significant differences between treatments at p < 0.05 calculated by Duncan’s multiple range tests. (TIF) [file pone.0171534.s003.tif]

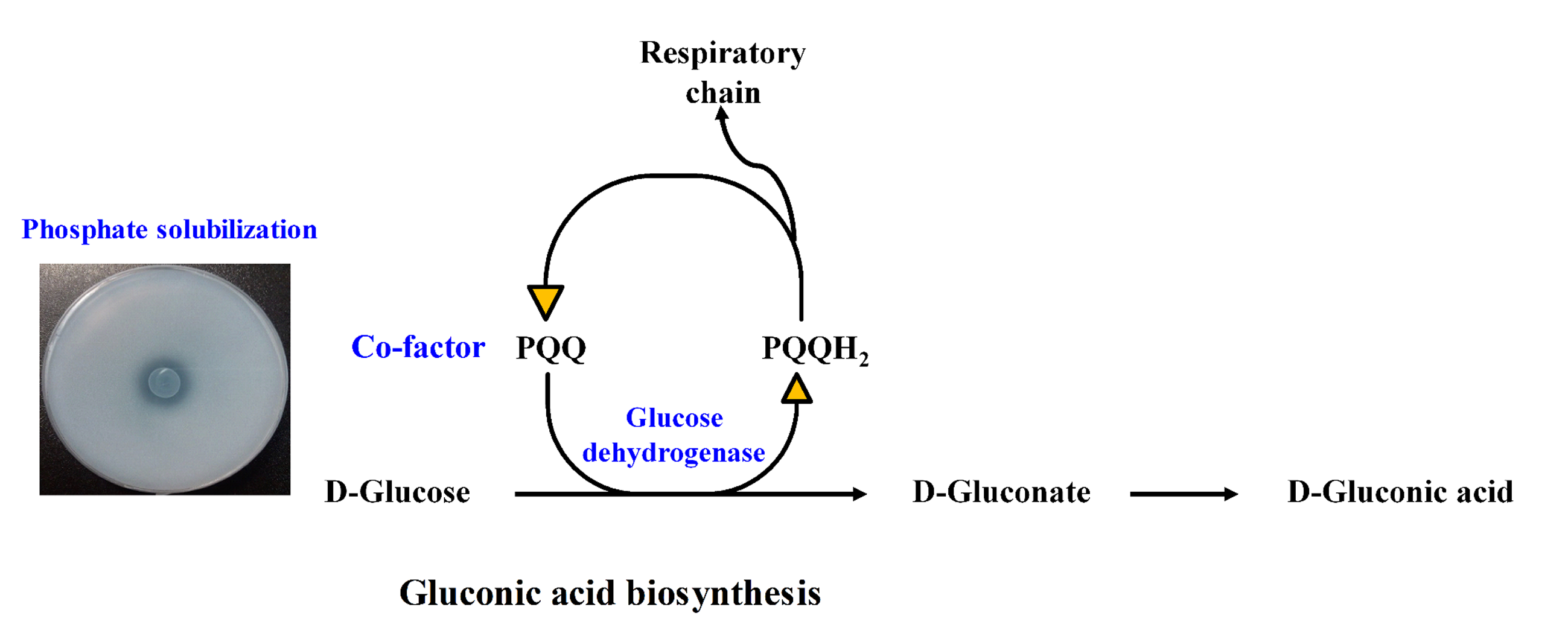

Supplement: S4 Fig — Gluconic acid is secreted by bacteria and plays a major role in phosphate solubilization [34]. The first enzyme of the gluconic acid pathway and cofactor (PQQ) are shown in blue. The gene encoding Glucose dehydrogenase and the pqqABCDEF operon were identified in the RSC-14 genome as locus-tags AN479_RS18465 and AN479_21790, AN479_RS21865, AN479_RS21870, AN479_RS21875, AN479_RS20540, AN479_RS21885, respectively. (TIF) [file pone.0171534.s004.tif]
